# Supplementary material for: Validation of the short Mood and Feelings Questionnaire in young adulthood
Source: J Affect Disord. 2021 Nov 1;294:883–8. doi: 10.1016/j.jad.2021.07.090 (PMC8411664; doi:10.1016/j.jad.2021.07.090)
Supplement: Supplementary file 1 [file mmc1.docx]

**Supplementary Material**

**Sensitivity Analysis: using complete sMFQ data**

For the primary analyses reported in the paper, where <10% items (1 item) were missing on the sMFQ, mean imputation was used to generate the missing value. A total of 111/4085 participants had one MFQ item missing, so mean imputation was used to generate one missing sMFQ value in 2.7% of the sample. Sensitivity analyses were undertaken including only those who had complete sMFQ data.

Including only individuals with complete sMFQ data in the analyses had very little effect on the results. The mean total sMFQ score for those with complete sMFQ data was 6.88 (SD=6.41, range 0-26), compared to 6.83 (SD= 6.40, range 0-26) for the sample with mean imputed sMFQ data. ROC analyses showed the AUC was 0.92 (95% CI= 0.90- 0.93) for both mean imputed and complete sMFQ samples. For those with complete sMFQ data, a cut point of ≥ 12 best balanced sensitivity and specificity (sensitivity=83.6%, specificity=85.23%) and a cut-point of ≥ 10 was most suitable when favouring sensitivity over specificity (sensitivity= 88.98%, specificity= 78.67%), the same cut-points as for the mean imputed sMFQ sample.

**Table S1: Association between demographic variables at baseline and presence of depression data at age 25 years**

|  | **Age 25: Depression data available**  **(n ≤ 4063)** | **Age 25: Depression data NOT available**  **(n ≤ 10,792)** | **Test statistic**  **(95% CI), p value** |
| --- | --- | --- | --- |
| **Gender, % male (n)** | 33.4 % (1356) | 57.8% (6236) | OR= 2.73 (2.53, 2.95),  p <0.001 |
| **Home owner, % (n)** | 83.9% (3074) | 69.1 % (6679) | OR= 0.43 (0.39, 0.47),  p <0.001 |
| **Maternal history of severe depression, % (n)** | 6.3 % (231) | 10.1 % (890) | OR= 1.66 (1.43, 1.93),  p <0.001 |
| **Maternal age, mean in years** | 29.2 years | 27.5 years | OR= 0.93 (0.92-0.94),  p <0.001 |
| **Maternal education, with A-levels or University, % (n)** | 48.0% (1746) | 30.0 % (2609) | OR= 0.47 (0.43, 0.50),  p <0.001 |

Note: presence of depression data= presence of both total MFQ score and DAWBA MDD data.
